# Retraction Note: Diagnostic power of ChatGPT 4 in distal radius fracture detection through wrist radiographs

**DOI:** 10.1007/s00402-024-05554-5

**Published:** 2024-09-14

**Authors:** Sinan Mert, Patrick Stoerzer, Johannes Brauer, Benedikt Fuchs, Elisabeth M. Haas-Lützenberger, Wolfram Demmer, Riccardo E. Giunta, Tim Nuernberger

**Affiliations:** Division of Hand, Plastic and Aesthetic Surgery, LMU University Hospital, LMU Munich, 80336 München, Germany

**Retraction Note: Archives of Orthopaedic and Trauma Surgery (2024) 144:2461–2467**

10.1007/s00402-024-05298-2

Authors have retracted this article because they lack proper permissions for X-rays and radiological reports employed in this study.

Wolfram Demmer, Sinan Mert, Benedikt Fuchs, Elisabeth M. Hass-Lützenberger, Riccardo E. Giunta, Patrick Stoerzer, and Johannes Brauer agree to this retraction. Tim Nuernberger has not responded to correspondence from the publisher about this retraction.

